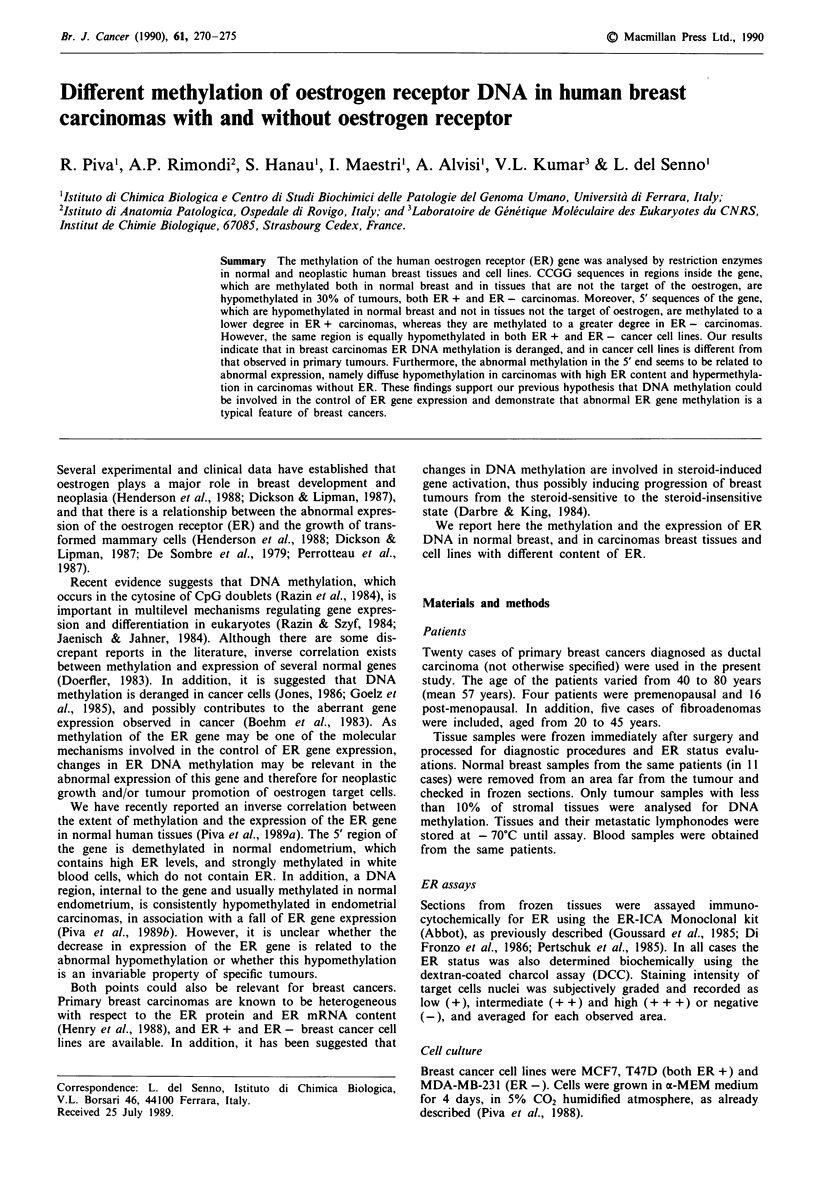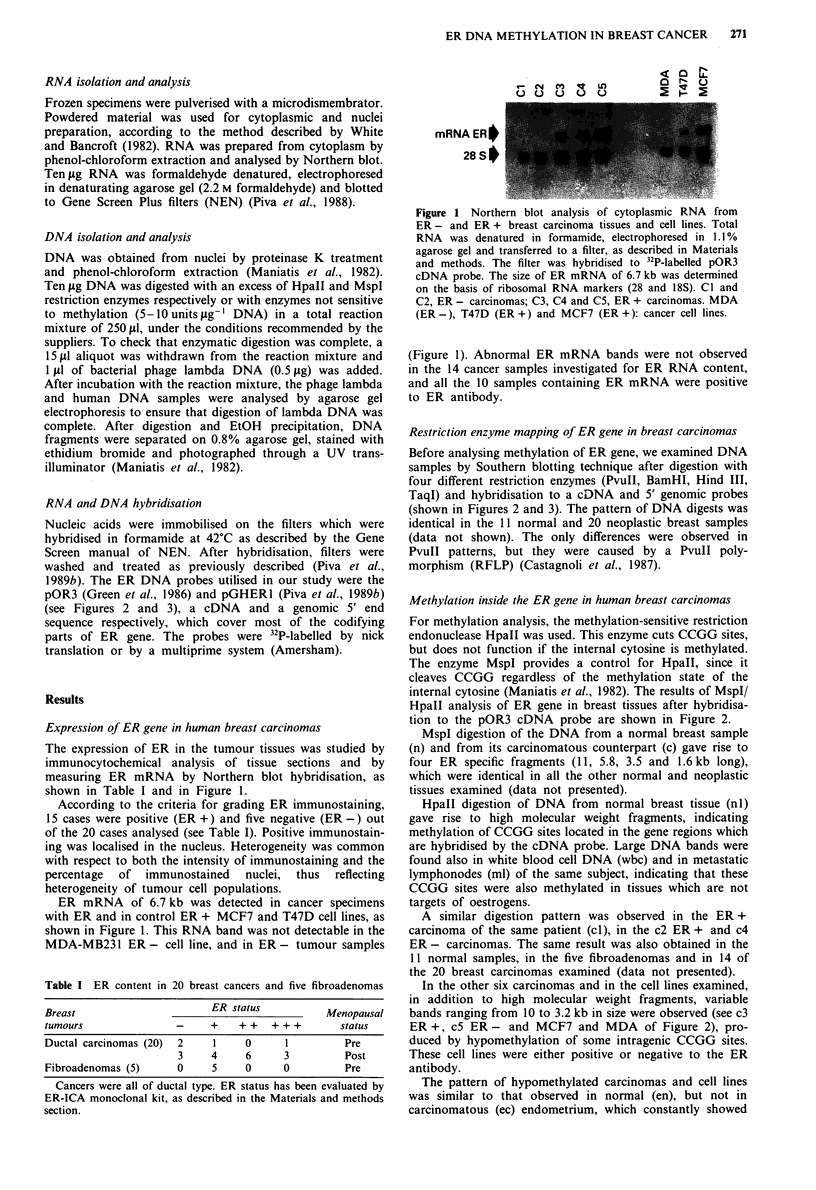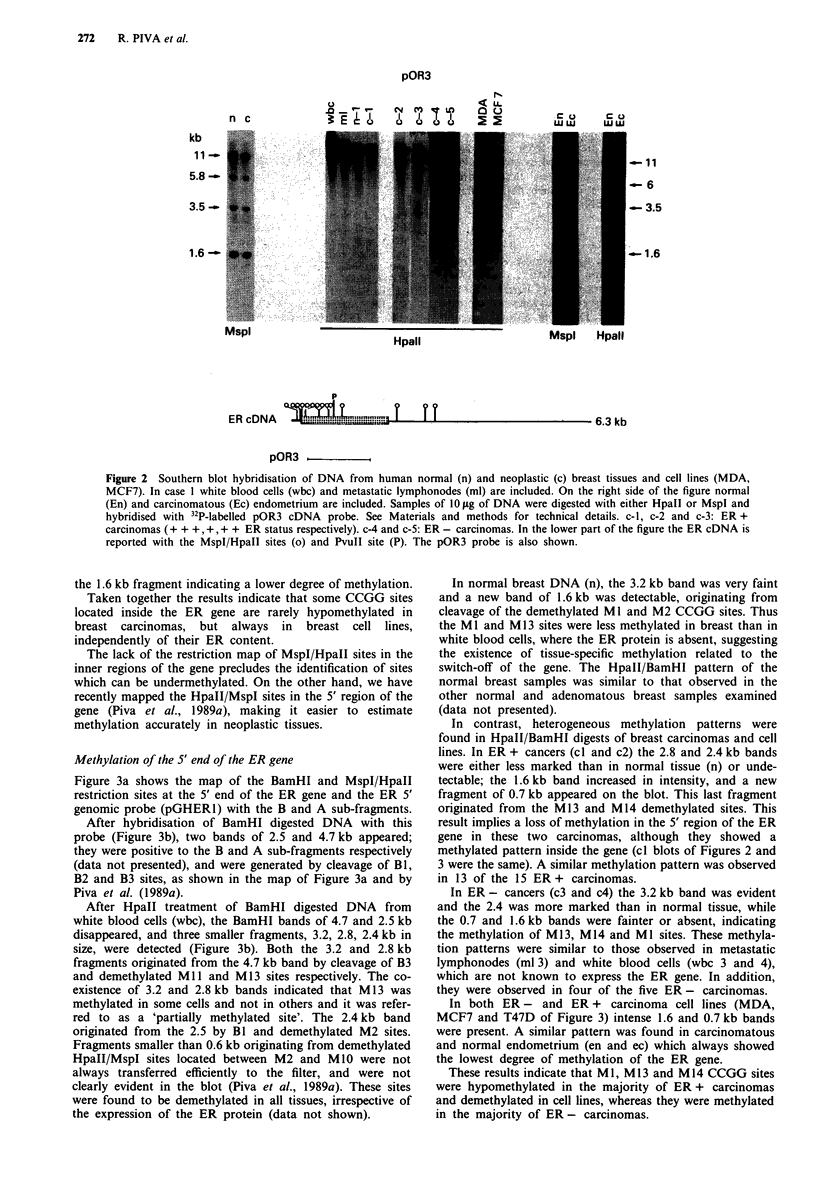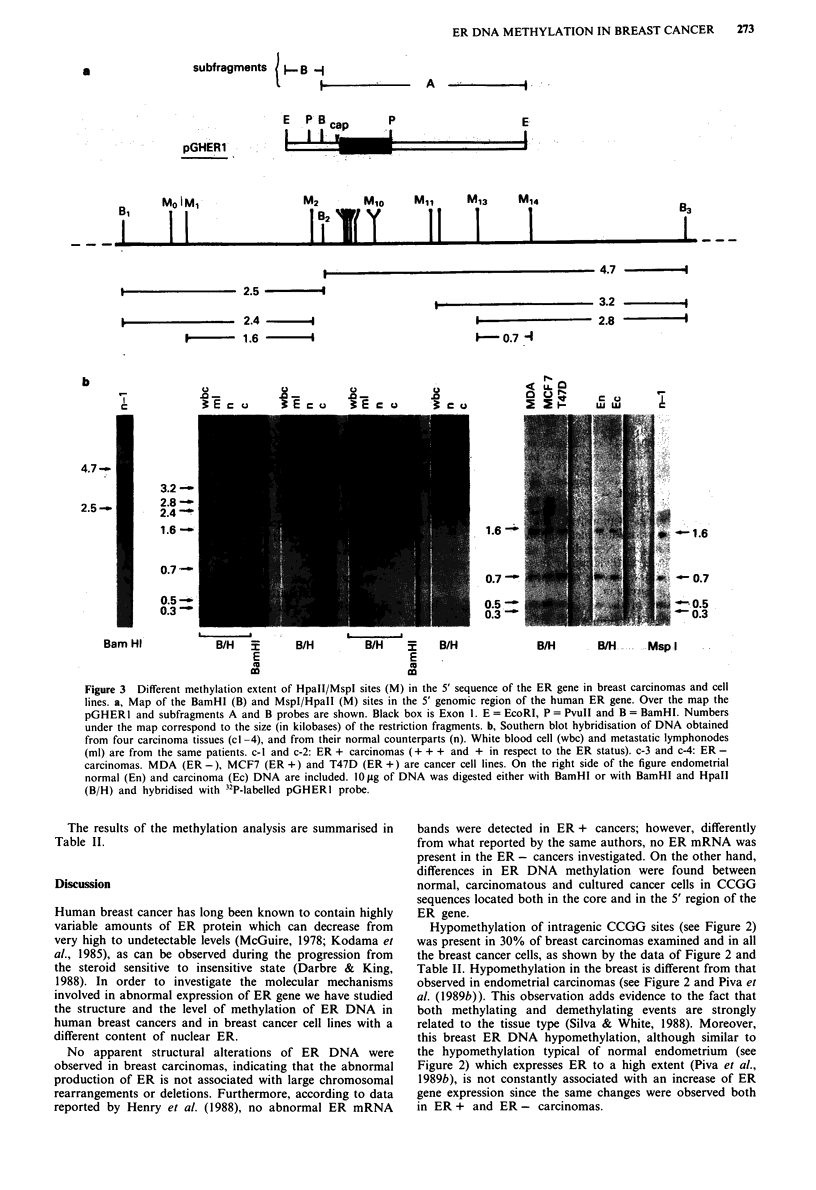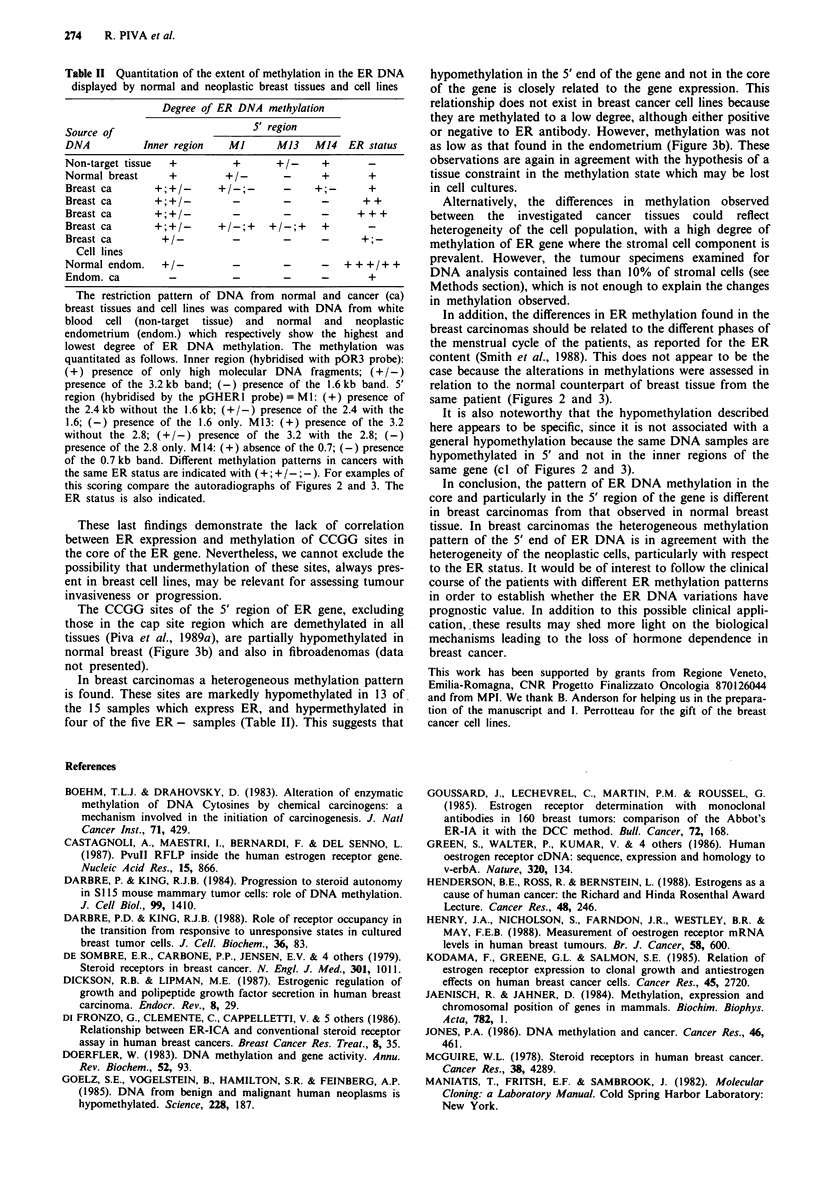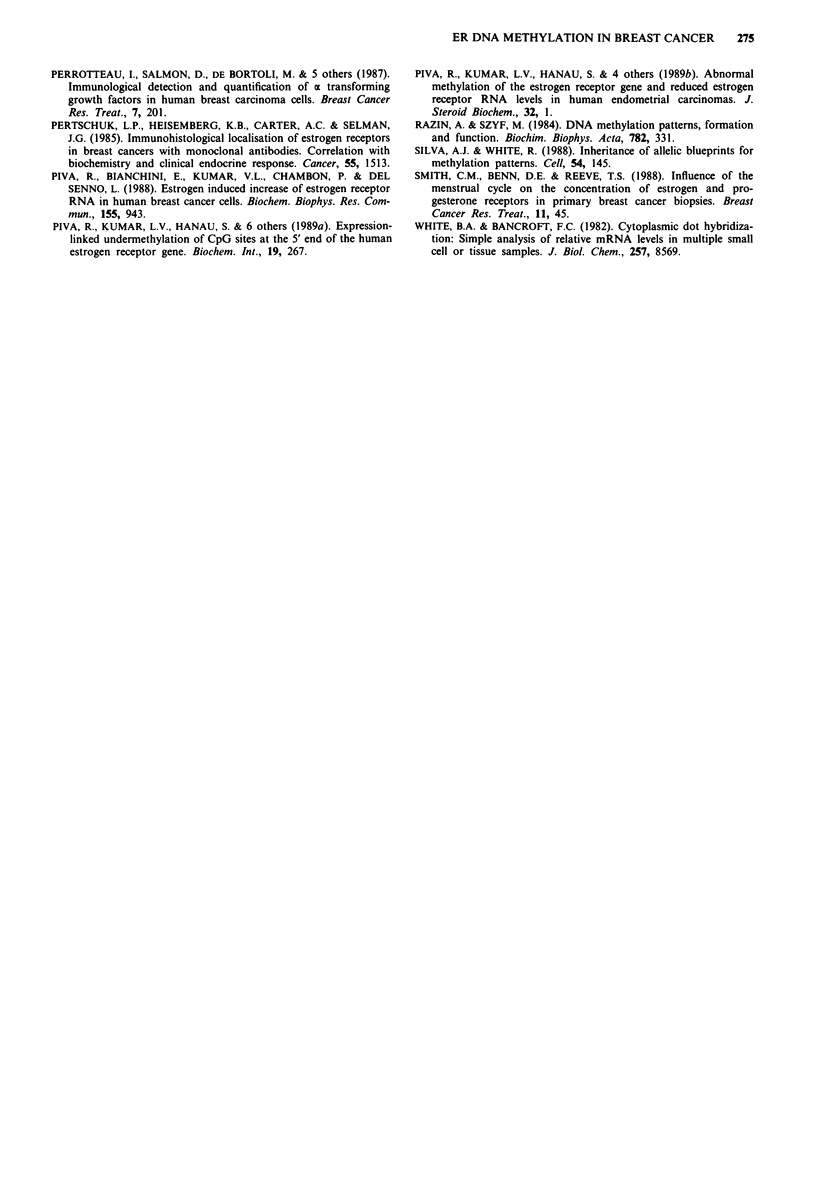# Different methylation of oestrogen receptor DNA in human breast carcinomas with and without oestrogen receptor.

**DOI:** 10.1038/bjc.1990.50

**Published:** 1990-02

**Authors:** R. Piva, A. P. Rimondi, S. Hanau, I. Maestri, A. Alvisi, V. L. Kumar, L. del Senno

**Affiliations:** Istituto di Chimica Biologica, Università di Ferrara, Italy.

## Abstract

**Images:**


*Br. J. Cancer* (1990), **61**, 270–275 © Macmillan Press Ltd., 1990

### Different methylation of oestrogen receptor DNA in human breast carcinomas with and without oestrogen receptor

R. Piva[1], A.P. Rimondi[2], S. Hanau[1], I. Maestri[1], A. Alvisi[1], V.L. Kumar[3] & L. del Senno[1]

[1]*Istituto di Chimica Biologica e Centro di Studi Biochimici delle Patologie del Genoma Umano, Università di Ferrara, Italy;* [2]*Istituto di Anatomia Patologica, Ospedale di Rovigo, Italy; and* [3]*Laboratoire de Génétique Moléculaire des Eukaryotes du CNRS, Institut de Chimie Biologique, 67085, Strasbourg Cedex, France.*

**Summary** The methylation of the human oestrogen receptor (ER) gene was analysed by restriction enzymes in normal and neoplastic human breast tissues and cell lines. CCGG sequences in regions inside the gene, which are methylated both in normal breast and in tissues that are not the target of the oestrogen, are hypomethylated in 30% of tumours, both ER + and ER − carcinomas. Moreover, 5′ sequences of the gene, which are hypomethylated in normal breast and not in tissues not the target of oestrogen, are methylated to a lower degree in ER + carcinomas, whereas they are methylated to a greater degree in ER − carcinomas. However, the same region is equally hypomethylated in both ER + and ER − cancer cell lines. Our results indicate that in breast carcinomas ER DNA methylation is deranged, and in cancer cell lines is different from that observed in primary tumours. Furthermore, the abnormal methylation in the 5′ end seems to be related to abnormal expression, namely diffuse hypomethylation in carcinomas with high ER content and hypermethylation in carcinomas without ER. These findings support our previous hypothesis that DNA methylation could be involved in the control of ER gene expression and demonstrate that abnormal ER gene methylation is a typical feature of breast cancers.

Several experimental and clinical data have established that oestrogen plays a major role in breast development and neoplasia (Henderson *et al.*, 1988; Dickson & Lipman, 1987), and that there is a relationship between the abnormal expression of the oestrogen receptor (ER) and the growth of transformed mammary cells (Henderson *et al.*, 1988; Dickson & Lipman, 1987; De Sombre *et al.*, 1979; Perrotteau *et al.*, 1987).

Recent evidence suggests that DNA methylation, which occurs in the cytosine of CpG doublets (Razin *et al.*, 1984), is important in multilevel mechanisms regulating gene expression and differentiation in eukaryotes (Razin & Szyf, 1984; Jaenisch & Jahner, 1984). Although there are some discrepant reports in the literature, inverse correlation exists between methylation and expression of several normal genes (Doerfler, 1983). In addition, it is suggested that DNA methylation is deranged in cancer cells (Jones, 1986; Goelz *et al.*, 1985), and possibly contributes to the aberrant gene expression observed in cancer (Boehm *et al.*, 1983). As methylation of the ER gene may be one of the molecular mechanisms involved in the control of ER gene expression, changes in ER DNA methylation may be relevant in the abnormal expression of this gene and therefore for neoplastic growth and/or tumour promotion of oestrogen target cells.

We have recently reported an inverse correlation between the extent of methylation and the expression of the ER gene in normal human tissues (Piva *et al.*, 1989*a*). The 5′ region of the gene is demethylated in normal endometrium, which contains high ER levels, and strongly methylated in white blood cells, which do not contain ER. In addition, a DNA region, internal to the gene and usually methylated in normal endometrium, is consistently hypomethylated in endometrial carcinomas, in association with a fall of ER gene expression (Piva *et al.*, 1989*b*). However, it is unclear whether the decrease in expression of the ER gene is related to the abnormal hypomethylation or whether this hypomethylation is an invariable property of specific tumours.

Both points could also be relevant for breast cancers. Primary breast carcinomas are known to be heterogeneous with respect to the ER protein and ER mRNA content (Henry *et al.*, 1988), and ER + and ER − breast cancer cell lines are available. In addition, it has been suggested that changes in DNA methylation are involved in steroid-induced gene activation, thus possibly inducing progression of breast tumours from the steroid-sensitive to the steroid-insensitive state (Darbre & King, 1984).

We report here the methylation and the expression of ER DNA in normal breast, and in carcinomas breast tissues and cell lines with different content of ER.

### Materials and methods

### *Patients*

Twenty cases of primary breast cancers diagnosed as ductal carcinoma (not otherwise specified) were used in the present study. The age of the patients varied from 40 to 80 years (mean 57 years). Four patients were premenopausal and 16 post-menopausal. In addition, five cases of fibroadenomas were included, aged from 20 to 45 years.

Tissue samples were frozen immediately after surgery and processed for diagnostic procedures and ER status evaluations. Normal breast samples from the same patients (in 11 cases) were removed from an area far from the tumour and checked in frozen sections. Only tumour samples with less than 10% of stromal tissues were analysed for DNA methylation. Tissues and their metastatic lymphonodes were stored at − 70°C until assay. Blood samples were obtained from the same patients.

### *ER assays*

Sections from frozen tissues were assayed immunocytochemically for ER using the ER-ICA Monoclonal kit (Abbot), as previously described (Goussard *et al.*, 1985; Di Fronzo *et al.*, 1986; Pertschuk *et al.*, 1985). In all cases the ER status was also determined biochemically using the dextran-coated charcol assay (DCC). Staining intensity of target cells nuclei was subjectively graded and recorded as low (+), intermediate (+ +) and high (+ + +) or negative (−), and averaged for each observed area.

### *Cell culture*

Breast cancer cell lines were MCF7, T47D (both ER +) and MDA-MB-231 (ER −). Cells were grown in α-MEM medium for 4 days, in 5% $CO_2$ humidified atmosphere, as already described (Piva *et al.*, 1988).

Correspondence: L. del Senno, Istituto di Chimica Biologica, V.L. Borsari 46, 44100 Ferrara, Italy.
Received 25 July 1989.

*RNA isolation and analysis*

Frozen specimens were pulverised with a microdismembrator. Powdered material was used for cytoplasmic and nuclei preparation, according to the method described by White and Bancroft (1982). RNA was prepared from cytoplasm by phenol-chloroform extraction and analysed by Northern blot. Ten μg RNA was formaldehyde denatured, electrophoresed in denaturating agarose gel (2.2 M formaldehyde) and blotted to Gene Screen Plus filters (NEN) (Piva *et al.*, 1988).

*DNA isolation and analysis*

DNA was obtained from nuclei by proteinase K treatment and phenol-chloroform extraction (Maniatis *et al.*, 1982). Ten μg DNA was digested with an excess of HpaII and MspI restriction enzymes respectively or with enzymes not sensitive to methylation (5–10 units $\mu g^{-1}$ DNA) in a total reaction mixture of 250 μl, under the conditions recommended by the suppliers. To check that enzymatic digestion was complete, a 15 μl aliquot was withdrawn from the reaction mixture and 1 μl of bacterial phage lambda DNA (0.5 μg) was added. After incubation with the reaction mixture, the phage lambda and human DNA samples were analysed by agarose gel electrophoresis to ensure that digestion of lambda DNA was complete. After digestion and EtOH precipitation, DNA fragments were separated on 0.8% agarose gel, stained with ethidium bromide and photographed through a UV trans-illuminator (Maniatis *et al.*, 1982).

*RNA and DNA hybridisation*

Nucleic acids were immobilised on the filters which were hybridised in formamide at 42°C as described by the Gene Screen manual of NEN. After hybridisation, filters were washed and treated as previously described (Piva *et al.*, 1989*b*). The ER DNA probes utilised in our study were the pOR3 (Green *et al.*, 1986) and pGHER1 (Piva *et al.*, 1989*b*) (see Figures 2 and 3), a cDNA and a genomic 5′ end sequence respectively, which cover most of the codifying parts of ER gene. The probes were $^{32}$P-labelled by nick translation or by a multiprime system (Amersham).

### Results

*Expression of ER gene in human breast carcinomas*

The expression of ER in the tumour tissues was studied by immunocytochemical analysis of tissue sections and by measuring ER mRNA by Northern blot hybridisation, as shown in Table I and in Figure 1.

According to the criteria for grading ER immunostaining, 15 cases were positive (ER +) and five negative (ER −) out of the 20 cases analysed (see Table I). Positive immunostaining was localised in the nucleus. Heterogeneity was common with respect to both the intensity of immunostaining and the percentage of immunostained nuclei, thus reflecting heterogeneity of tumour cell populations.

ER mRNA of 6.7 kb was detected in cancer specimens with ER and in control ER + MCF7 and T47D cell lines, as shown in Figure 1. This RNA band was not detectable in the MDA-MB231 ER − cell line, and in ER − tumour samples

**Table I** ER content in 20 breast cancers and five fibroadenomas

| Breast tumours | ER status − | + | + + | + + + | Menopausal status |
|---|---|---|---|---|---|
| Ductal carcinomas (20) | 2 | 1 | 0 | 1 | Pre |
| | 3 | 4 | 6 | 3 | Post |
| Fibroadenomas (5) | 0 | 5 | 0 | 0 | Pre |

Cancers were all of ductal type. ER status has been evaluated by ER-ICA monoclonal kit, as described in the Materials and methods section.

**Figure 1** Northern blot analysis of cytoplasmic RNA from ER − and ER + breast carcinoma tissues and cell lines. Total RNA was denatured in formamide, electrophoresed in 1.1% agarose gel and transferred to a filter, as described in Materials and methods. The filter was hybridised to $^{32}$P-labelled pOR3 cDNA probe. The size of ER mRNA of 6.7 kb was determined on the basis of ribosomal RNA markers (28 and 18S). C1 and C2, ER − carcinomas; C3, C4 and C5, ER + carcinomas. MDA (ER −), T47D (ER +) and MCF7 (ER +): cancer cell lines.

(Figure 1). Abnormal ER mRNA bands were not observed in the 14 cancer samples investigated for ER RNA content, and all the 10 samples containing ER mRNA were positive to ER antibody.

*Restriction enzyme mapping of ER gene in breast carcinomas*

Before analysing methylation of ER gene, we examined DNA samples by Southern blotting technique after digestion with four different restriction enzymes (PvuII, BamHI, Hind III, TaqI) and hybridisation to a cDNA and 5′ genomic probes (shown in Figures 2 and 3). The pattern of DNA digests was identical in the 11 normal and 20 neoplastic breast samples (data not shown). The only differences were observed in PvuII patterns, but they were caused by a PvuII polymorphism (RFLP) (Castagnoli *et al.*, 1987).

*Methylation inside the ER gene in human breast carcinomas*

For methylation analysis, the methylation-sensitive restriction endonuclease HpaII was used. This enzyme cuts CCGG sites, but does not function if the internal cytosine is methylated. The enzyme MspI provides a control for HpaII, since it cleaves CCGG regardless of the methylation state of the internal cytosine (Maniatis *et al.*, 1982). The results of MspI/HpaII analysis of ER gene in breast tissues after hybridisation to the pOR3 cDNA probe are shown in Figure 2.

MspI digestion of the DNA from a normal breast sample (n) and from its carcinomatous counterpart (c) gave rise to four ER specific fragments (11, 5.8, 3.5 and 1.6 kb long), which were identical in all the other normal and neoplastic tissues examined (data not presented).

HpaII digestion of DNA from normal breast tissue (n1) gave rise to high molecular weight fragments, indicating methylation of CCGG sites located in the gene regions which are hybridised by the cDNA probe. Large DNA bands were found also in white blood cell DNA (wbc) and in metastatic lymphonodes (ml) of the same subject, indicating that these CCGG sites were also methylated in tissues which are not targets of oestrogens.

A similar digestion pattern was observed in the ER + carcinoma of the same patient (c1), in the c2 ER + and c4 ER − carcinomas. The same result was also obtained in the 11 normal samples, in the five fibroadenomas and in 14 of the 20 breast carcinomas examined (data not presented).

In the other six carcinomas and in the cell lines examined, in addition to high molecular weight fragments, variable bands ranging from 10 to 3.2 kb in size were observed (see c3 ER +, c5 ER − and MCF7 and MDA of Figure 2), produced by hypomethylation of some intragenic CCGG sites. These cell lines were either positive or negative to the ER antibody.

The pattern of hypomethylated carcinomas and cell lines was similar to that observed in normal (en), but not in carcinomatous (ec) endometrium, which constantly showed

**Figure 2** Southern blot hybridisation of DNA from human normal (n) and neoplastic (c) breast tissues and cell lines (MDA, MCF7). In case 1 white blood cells (wbc) and metastatic lymphonodes (ml) are included. On the right side of the figure normal (En) and carcinomatous (Ec) endometrium are included. Samples of 10 μg of DNA were digested with either HpaII or MspI and hybridised with $^{32}$P-labelled pOR3 cDNA probe. See Materials and methods for technical details. c-1, c-2 and c-3: ER + carcinomas (+ + +, +, + + ER status respectively). c-4 and c-5: ER – carcinomas. In the lower part of the figure the ER cDNA is reported with the MspI/HpaII sites (o) and PvuII site (P). The pOR3 probe is also shown.

the 1.6 kb fragment indicating a lower degree of methylation.

Taken together the results indicate that some CCGG sites located inside the ER gene are rarely hypomethylated in breast carcinomas, but always in breast cell lines, independently of their ER content.

The lack of the restriction map of MspI/HpaII sites in the inner regions of the gene precludes the identification of sites which can be undermethylated. On the other hand, we have recently mapped the HpaII/MspI sites in the 5′ region of the gene (Piva *et al.*, 1989*a*), making it easier to estimate methylation accurately in neoplastic tissues.

*Methylation of the 5′ end of the ER gene*

Figure 3a shows the map of the BamHI and MspI/HpaII restriction sites at the 5′ end of the ER gene and the ER 5′ genomic probe (pGHER1) with the B and A sub-fragments.

After hybridisation of BamHI digested DNA with this probe (Figure 3b), two bands of 2.5 and 4.7 kb appeared; they were positive to the B and A sub-fragments respectively (data not presented), and were generated by cleavage of B1, B2 and B3 sites, as shown in the map of Figure 3a and by Piva *et al.* (1989*a*).

After HpaII treatment of BamHI digested DNA from white blood cells (wbc), the BamHI bands of 4.7 and 2.5 kb disappeared, and three smaller fragments, 3.2, 2.8, 2.4 kb in size, were detected (Figure 3b). Both the 3.2 and 2.8 kb fragments originated from the 4.7 kb band by cleavage of B3 and demethylated M11 and M13 sites respectively. The co-existence of 3.2 and 2.8 kb bands indicated that M13 was methylated in some cells and not in others and it was referred to as a 'partially methylated site'. The 2.4 kb band originated from the 2.5 by B1 and demethylated M2 sites. Fragments smaller than 0.6 kb originating from demethylated HpaII/MspI sites located between M2 and M10 were not always transferred efficiently to the filter, and were not clearly evident in the blot (Piva *et al.*, 1989*a*). These sites were found to be demethylated in all tissues, irrespective of the expression of the ER protein (data not shown).

In normal breast DNA (n), the 3.2 kb band was very faint and a new band of 1.6 kb was detectable, originating from cleavage of the demethylated M1 and M2 CCGG sites. Thus the M1 and M13 sites were less methylated in breast than in white blood cells, where the ER protein is absent, suggesting the existence of tissue-specific methylation related to the switch-off of the gene. The HpaII/BamHI pattern of the normal breast samples was similar to that observed in the other normal and adenomatous breast samples examined (data not presented).

In contrast, heterogeneous methylation patterns were found in HpaII/BamHI digests of breast carcinomas and cell lines. In ER + cancers (c1 and c2) the 2.8 and 2.4 kb bands were either less marked than in normal tissue (n) or undetectable; the 1.6 kb band increased in intensity, and a new fragment of 0.7 kb appeared on the blot. This last fragment originated from the M13 and M14 demethylated sites. This result implies a loss of methylation in the 5′ region of the ER gene in these two carcinomas, although they showed a methylated pattern inside the gene (c1 blots of Figures 2 and 3 were the same). A similar methylation pattern was observed in 13 of the 15 ER + carcinomas.

In ER – cancers (c3 and c4) the 3.2 kb band was evident and the 2.4 was more marked than in normal tissue, while the 0.7 and 1.6 kb bands were fainter or absent, indicating the methylation of M13, M14 and M1 sites. These methylation patterns were similar to those observed in metastatic lymphonodes (ml 3) and white blood cells (wbc 3 and 4), which are not known to express the ER gene. In addition, they were observed in four of the five ER – carcinomas.

In both ER – and ER + carcinoma cell lines (MDA, MCF7 and T47D of Figure 3) intense 1.6 and 0.7 kb bands were present. A similar pattern was found in carcinomatous and normal endometrium (en and ec) which always showed the lowest degree of methylation of the ER gene.

These results indicate that M1, M13 and M14 CCGG sites were hypomethylated in the majority of ER + carcinomas and demethylated in cell lines, whereas they were methylated in the majority of ER – carcinomas.

**Figure 3** Different methylation extent of HpaII/MspI sites (M) in the 5′ sequence of the ER gene in breast carcinomas and cell lines. **a**, Map of the BamHI (B) and MspI/HpaII (M) sites in the 5′ genomic region of the human ER gene. Over the map the pGHER1 and subfragments A and B probes are shown. Black box is Exon 1. E = EcoRI, P = PvuII and B = BamHI. Numbers under the map correspond to the size (in kilobases) of the restriction fragments. **b**, Southern blot hybridisation of DNA obtained from four carcinoma tissues (c1–4), and from their normal counterparts (n). White blood cell (wbc) and metastatic lymphonodes (ml) are from the same patients. c-1 and c-2: ER + carcinomas (+ + + and + in respect to the ER status). c-3 and c-4: ER − carcinomas. MDA (ER −), MCF7 (ER +) and T47D (ER +) are cancer cell lines. On the right side of the figure endometrial normal (En) and carcinoma (Ec) DNA are included. 10 µg of DNA was digested either with BamHI or with BamHI and HpaII (B/H) and hybridised with $^{32}$P-labelled pGHER1 probe.

The results of the methylation analysis are summarised in Table II.

### Discussion

Human breast cancer has long been known to contain highly variable amounts of ER protein which can decrease from very high to undetectable levels (McGuire, 1978; Kodama *et al.*, 1985), as can be observed during the progression from the steroid sensitive to insensitive state (Darbre & King, 1988). In order to investigate the molecular mechanisms involved in abnormal expression of ER gene we have studied the structure and the level of methylation of ER DNA in human breast cancers and in breast cancer cell lines with a different content of nuclear ER.

No apparent structural alterations of ER DNA were observed in breast carcinomas, indicating that the abnormal production of ER is not associated with large chromosomal rearrangements or deletions. Furthermore, according to data reported by Henry *et al.* (1988), no abnormal ER mRNA bands were detected in ER + cancers; however, differently from what reported by the same authors, no ER mRNA was present in the ER − cancers investigated. On the other hand, differences in ER DNA methylation were found between normal, carcinomatous and cultured cancer cells in CCGG sequences located both in the core and in the 5′ region of the ER gene.

Hypomethylation of intragenic CCGG sites (see Figure 2) was present in 30% of breast carcinomas examined and in all the breast cancer cells, as shown by the data of Figure 2 and Table II. Hypomethylation in the breast is different from that observed in endometrial carcinomas (see Figure 2 and Piva *et al.* (1989*b*)). This observation adds evidence to the fact that both methylating and demethylating events are strongly related to the tissue type (Silva & White, 1988). Moreover, this breast ER DNA hypomethylation, although similar to the hypomethylation typical of normal endometrium (see Figure 2) which expresses ER to a high extent (Piva *et al.*, 1989*b*), is not constantly associated with an increase of ER gene expression since the same changes were observed both in ER + and ER − carcinomas.

**Table II** Quantitation of the extent of methylation in the ER DNA displayed by normal and neoplastic breast tissues and cell lines

| | *Degree of ER DNA methylation* | | | | |
|---|---|---|---|---|---|
| | | *5′ region* | | | |
| *Source of DNA* | *Inner region* | *M1* | *M13* | *M14* | *ER status* |
| Non-target tissue | + | + | +/− | + | − |
| Normal breast | + | +/− | − | + | + |
| Breast ca | +;+/− | +/−;− | − | +;− | + |
| Breast ca | +;+/− | − | − | − | ++ |
| Breast ca | +;+/− | − | − | − | +++ |
| Breast ca | +;+/− | +/−;+ | +/−;+ | + | − |
| Breast ca Cell lines | +/− | − | − | − | +;− |
| Normal endom. | +/− | − | − | − | +++/++ |
| Endom. ca | − | − | − | − | + |

The restriction pattern of DNA from normal and cancer (ca) breast tissues and cell lines was compared with DNA from white blood cell (non-target tissue) and normal and neoplastic endometrium (endom.) which respectively show the highest and lowest degree of ER DNA methylation. The methylation was quantitated as follows. Inner region (hybridised with pOR3 probe): (+) presence of only high molecular DNA fragments; (+/−) presence of the 3.2 kb band; (−) presence of the 1.6 kb band. 5′ region (hybridised by the pGHER1 probe) = M1: (+) presence of the 2.4 kb without the 1.6 kb; (+/−) presence of the 2.4 with the 1.6; (−) presence of the 1.6 only. M13: (+) presence of the 3.2 without the 2.8; (+/−) presence of the 3.2 with the 2.8; (−) presence of the 2.8 only. M14: (+) absence of the 0.7; (−) presence of the 0.7 kb band. Different methylation patterns in cancers with the same ER status are indicated with (+;+/−;−). For examples of this scoring compare the autoradiographs of Figures 2 and 3. The ER status is also indicated.

These last findings demonstrate the lack of correlation between ER expression and methylation of CCGG sites in the core of the ER gene. Nevertheless, we cannot exclude the possibility that undermethylation of these sites, always present in breast cell lines, may be relevant for assessing tumour invasiveness or progression.

The CCGG sites of the 5′ region of ER gene, excluding those in the cap site region which are demethylated in all tissues (Piva *et al.*, 1989*a*), are partially hypomethylated in normal breast (Figure 3b) and also in fibroadenomas (data not presented).

In breast carcinomas a heterogeneous methylation pattern is found. These sites are markedly hypomethylated in 13 of the 15 samples which express ER, and hypermethylated in four of the five ER − samples (Table II). This suggests that hypomethylation in the 5′ end of the gene and not in the core of the gene is closely related to the gene expression. This relationship does not exist in breast cancer cell lines because they are methylated to a low degree, although either positive or negative to ER antibody. However, methylation was not as low as that found in the endometrium (Figure 3b). These observations are again in agreement with the hypothesis of a tissue constraint in the methylation state which may be lost in cell cultures.

Alternatively, the differences in methylation observed between the investigated cancer tissues could reflect heterogeneity of the cell population, with a high degree of methylation of ER gene where the stromal cell component is prevalent. However, the tumour specimens examined for DNA analysis contained less than 10% of stromal cells (see Methods section), which is not enough to explain the changes in methylation observed.

In addition, the differences in ER methylation found in the breast carcinomas should be related to the different phases of the menstrual cycle of the patients, as reported for the ER content (Smith *et al.*, 1988). This does not appear to be the case because the alterations in methylations were assessed in relation to the normal counterpart of breast tissue from the same patient (Figures 2 and 3).

It is also noteworthy that the hypomethylation described here appears to be specific, since it is not associated with a general hypomethylation because the same DNA samples are hypomethylated in 5′ and not in the inner regions of the same gene (cl of Figures 2 and 3).

In conclusion, the pattern of ER DNA methylation in the core and particularly in the 5′ region of the gene is different in breast carcinomas from that observed in normal breast tissue. In breast carcinomas the heterogeneous methylation pattern of the 5′ end of ER DNA is in agreement with the heterogeneity of the neoplastic cells, particularly with respect to the ER status. It would be of interest to follow the clinical course of the patients with different ER methylation patterns in order to establish whether the ER DNA variations have prognostic value. In addition to this possible clinical application, these results may shed more light on the biological mechanisms leading to the loss of hormone dependence in breast cancer.

This work has been supported by grants from Regione Veneto, Emilia-Romagna, CNR Progetto Finalizzato Oncologia 870126044 and from MPI. We thank B. Anderson for helping us in the preparation of the manuscript and I. Perrotteau for the gift of the breast cancer cell lines.

### References

BOEHM, T.L.J. & DRAHOVSKY, D. (1983). Alteration of enzymatic methylation of DNA Cytosines by chemical carcinogens: a mechanism involved in the initiation of carcinogenesis. *J. Natl Cancer Inst.*, **71**, 429.

CASTAGNOLI, A., MAESTRI, I., BERNARDI, F. & DEL SENNO, L. (1987). PvuII RFLP inside the human estrogen receptor gene. *Nucleic Acid Res.*, **15**, 866.

DARBRE, P. & KING, R.J.B. (1984). Progression to steroid autonomy in S115 mouse mammary tumor cells: role of DNA methylation. *J. Cell Biol.*, **99**, 1410.

DARBRE, P.D. & KING, R.J.B. (1988). Role of receptor occupancy in the transition from responsive to unresponsive states in cultured breast tumor cells. *J. Cell. Biochem.*, **36**, 83.

DE SOMBRE, E.R., CARBONE, P.P., JENSEN, E.V. & 4 others (1979). Steroid receptors in breast cancer. *N. Engl. J. Med.*, **301**, 1011.

DICKSON, R.B. & LIPMAN, M.E. (1987). Estrogenic regulation of growth and polipeptide growth factor secretion in human breast carcinoma. *Endocr. Rev.*, **8**, 29.

DI FRONZO, G., CLEMENTE, C., CAPPELLETTI, V. & 5 others (1986). Relationship between ER-ICA and conventional steroid receptor assay in human breast cancers. *Breast Cancer Res. Treat.*, **8**, 35.

DOERFLER, W. (1983). DNA methylation and gene activity. *Annu. Rev. Biochem.*, **52**, 93.

GOELZ, S.E., VOGELSTEIN, B., HAMILTON, S.R. & FEINBERG, A.P. (1985). DNA from benign and malignant human neoplasms is hypomethylated. *Science*, **228**, 187.

GOUSSARD, J., LECHEVREL, C., MARTIN, P.M. & ROUSSEL, G. (1985). Estrogen receptor determination with monoclonal antibodies in 160 breast tumors: comparison of the Abbot's ER-IA it with the DCC method. *Bull. Cancer*, **72**, 168.

GREEN, S., WALTER, P., KUMAR, V. & 4 others (1986). Human oestrogen receptor cDNA: sequence, expression and homology to v-erbA. *Nature*, **320**, 134.

HENDERSON, B.E., ROSS, R. & BERNSTEIN, L. (1988). Estrogens as a cause of human cancer: the Richard and Hinda Rosenthal Award Lecture. *Cancer Res.*, **48**, 246.

HENRY, J.A., NICHOLSON, S., FARNDON, J.R., WESTLEY, B.R. & MAY, F.E.B. (1988). Measurement of oestrogen receptor mRNA levels in human breast tumours. *Br. J. Cancer*, **58**, 600.

KODAMA, F., GREENE, G.L. & SALMON, S.E. (1985). Relation of estrogen receptor expression to clonal growth and antiestrogen effects on human breast cancer cells. *Cancer Res.*, **45**, 2720.

JAENISCH, R. & JAHNER, D. (1984). Methylation, expression and chromosomal position of genes in mammals. *Biochim. Biophys. Acta*, **782**, 1.

JONES, P.A. (1986). DNA methylation and cancer. *Cancer Res.*, **46**, 461.

MCGUIRE, W.L. (1978). Steroid receptors in human breast cancer. *Cancer Res.*, **38**, 4289.

MANIATIS, T., FRITSH, E.F. & SAMBROOK, J. (1982). *Molecular Cloning: a Laboratory Manual.* Cold Spring Harbor Laboratory: New York.

PERROTTEAU, I., SALMON, D., DE BORTOLI, M. & 5 others (1987). Immunological detection and quantification of α transforming growth factors in human breast carcinoma cells. *Breast Cancer Res. Treat.*, **7**, 201.

PERTSCHUK, L.P., HEISEMBERG, K.B., CARTER, A.C. & SELMAN, J.G. (1985). Immunohistological localisation of estrogen receptors in breast cancers with monoclonal antibodies. Correlation with biochemistry and clinical endocrine response. *Cancer*, **55**, 1513.

PIVA, R., BIANCHINI, E., KUMAR, V.L., CHAMBON, P. & DEL SENNO, L. (1988). Estrogen induced increase of estrogen receptor RNA in human breast cancer cells. *Biochem. Biophys. Res. Commun.*, **155**, 943.

PIVA, R., KUMAR, L.V., HANAU, S. & 6 others (1989*a*). Expression-linked undermethylation of CpG sites at the 5′ end of the human estrogen receptor gene. *Biochem. Int.*, **19**, 267.

PIVA, R., KUMAR, L.V., HANAU, S. & 4 others (1989*b*). Abnormal methylation of the estrogen receptor gene and reduced estrogen receptor RNA levels in human endometrial carcinomas. *J. Steroid Biochem.*, **32**, 1.

RAZIN, A. & SZYF, M. (1984). DNA methylation patterns, formation and function. *Biochim. Biophys. Acta*, **782**, 331.

SILVA, A.J. & WHITE, R. (1988). Inheritance of allelic blueprints for methylation patterns. *Cell*, **54**, 145.

SMITH, C.M., BENN, D.E. & REEVE, T.S. (1988). Influence of the menstrual cycle on the concentration of estrogen and progesterone receptors in primary breast cancer biopsies. *Breast Cancer Res. Treat.*, **11**, 45.

WHITE, B.A. & BANCROFT, F.C. (1982). Cytoplasmic dot hybridization: Simple analysis of relative mRNA levels in multiple small cell or tissue samples. *J. Biol. Chem.*, **257**, 8569.